# Myo-inositol effect on pregnancy outcomes in infertile women undergoing in vitro fertilization/intracytoplasmic sperm injection: A double-blind RCT

**DOI:** 10.18502/ijrm.v20i8.11753

**Published:** 2022-09-06

**Authors:** Fariba Seyedoshohadaei, Shahin Abbasi, Masoumeh Rezaie, Azra Allahvaisi, Mohammad Jafar Rezaie, Nasrin Soufizadeh, Khaled Rahmani

**Affiliations:** ^1^Department of Obstetrics and Gynecology, Faculty of Medicine, Kurdistan University of Medical Sciences, Sanandaj, Iran.; ^2^Infertility Treatment Center of Besat Hospital, Kurdistan University of Medical Sciences, Sanandaj, Iran.; ^3^Department of Anatomy, School of Medicine, Kurdistan University of Medical Sciences, Kurdistan, Iran.; ^4^Liver and Digestive Research Center, Research Institute for Health Development, Kurdistan University of Medical Sciences, Sanandaj, Iran.

**Keywords:** Infertility, In vitro fertilization, Intracytoplasmic sperm injection, Myo-inositol.

## Abstract

**Background:**

Myo-inositol is an intracellular mediator which is involved in various aspects of reproduction in women.

**Objective:**

This study aimed to evaluate the impact of Myo-inositol on the outcomes of in vitro fertilization (IVF)/intracytoplasmic sperm injection (ICSI) cycles in infertile women.

**Materials and Methods:**

This double-blind randomized controlled trial was conducted on 70 infertile women referred to the Infertility Treatment Center, Besat hospital, Sanandaj, Iran from May 2019 to September 2019 for IVF/ICSI cycles. The participants were randomly divided into 2 intervention (n = 36) and control (n = 34) groups. The intervention group received 2000 mg of Myo-inositol and 200 mcg folic acid twice a day for 2 months and the control group received 200 mcg of folic acid twice a day for 2 months in the IVF/ICSI cycles (from the third day of cycle until the end of the second month). Finally, the number of oocytes, the quality of embryos, and the IVF/ICSI outcomes were compared between the 2 groups.

**Results:**

The mean numbers of oocytes, MII oocytes, and 2 pronuclear embryos were significantly higher in the intervention group than the control group. Also, the clinical pregnancy and live birth rates in the intervention group were significantly higher than in the controls (p = 0.04).

**Conclusion:**

The administration of Myo-inositol may increase clinical pregnancy and live birth rates by increasing the number of total and meiosis II oocytes in infertile women undergoing IVF/ICSI.

## 1. Introduction

Nowadays assisted reproductive techniques (ARTs) are used to achieve fertility in infertile people. In vitro fertilization (IVF) and intracytoplasmic sperm injection (ICSI) are effective ARTs (1). IVF and ICSI are the most appropriate treatments in infertility clinics. However, one of the most important problems of these methods is the low rate of pregnancy success (2).

Despite significant advances in ARTs, there are still problems in achieving desirable results through IVF and ICSI techniques. Using supplements in ARTs can be very helpful in increasing pregnancy rates (3).

The balance between the production and clearance of reactiveoxygen species is critical to the health of the female reproductive system. Antioxidants are compounds that can decrease oxidative damage in infertile women (4).

Myo-inositol is a C6 sugar alcohol belonging to the B-group vitamins (5). Myo-inositol plays a key role in all aspects of cell physiology including lipid synthesis, cell morphogenesis, cell membrane structures, and cell membrane functions (6, 7).

Studies on inositol and its isoforms (particularly Myo-inositol) in IVF have demonstrated that this molecule reduces insulin resistance and improves ovarian function, oocyte quality, and embryo pregnancy rates (8). The inositol 1,4,5-triphosphate receptor channel is found in mammalian cells which are intracellular mediators for the release of calcium (9, 10). Evidence suggests that this receptor channel has a key role in regulating calcium signaling in the oocytes of mammals (11). The mechanisms of calcium release during oogenesis play an important role in the success of fertilization. Several reports point to the possible role of inositol phospholipids-calcium in oocyte development (12, 13).

The goal of this research was to assess the impact of Myo-inositol on pregnancy success in infertile women candidates for IVF and ICSI in the Infertility Treatment Center, Besat hospital, Kurdistan University of Medical Sciences, Sanandaj, Iran.

## 2. Materials and Methods

### Study design, participants, and setting

This double-blind, randomized clinical trial was conducted in 70 infertile women referred to the Infertility Treatment Center, Besat hospital, Sanandaj, Iran, from May 2019 to September 2019 for IVF/ICSI cycles. The women were randomly divided into 2 intervention and control groups using permuted block randomization (n = 30/each).

Our inclusion criteria were age 20-40 yr, infertility for at least 1 year, candidates for IVF/ICSI treatments, not used contraception methods for at least 1 year ago, agonist cycles, and having a regular menstrual cycle (24-35 days). All women with any underling diseses such as metabolic or endocrine disorders, uterine anomaly, frozen embryo transfer cycle and history of recurrent miscarriages were excluded.

The intervention group received 2000 mg of Myo-inositol (Inofolic sachet, Lo. Li Pharma. Tehran, Iran) plus 200 mcg of folic acid (Raha pharmaceutical Co., Isfahan, Iran) twice a day for 2 months from the third day of cycle until the end of the second month. The control group received 200 mcg of folic acid (Raha pharmaceutical Co., Isfahan, Iran) twice a day for 2 months from the 3^rd^ day of cycle until the end of the 2^nd^ month.

### The stimulation protocol and the IVF/ICSI cycles

The ovarian hyperstimulation protocol was performed for all the participants in the studied groups according to the gonadotropin-releasing hormone agonist protocol.

Low dose oral contraceptive pills were administered from the 3 ${}^{\text{rd}}$ day to the 23 ${}^{\text{rd}}$ day of the 1 ${}^{\text{st}}$ menstrual cycle. Sub-cutaneous injections of the gonadotropin-releasing hormone analog (Buserelin, Cinna Fact, Cinna Gen, Iran) were started on day 21 of the 1 ${}^{\text{st}}$ menstrual cycle with a dose of 0.5 mg and continued until the 2 ${}^{\text{nd}}$ day of the 2 ${}^{\text{nd}}$ cycle. The dose was gradually reduced to 0.25 mg until oocyte retrieval. Ovulation induction was started from the 3 ${}^{\text{rd}}$ day of the 2 ${}^{\text{nd}}$ cycle with a recombinant follicle-stimulating hormone (Cinnal-F, Cinna Gen, Iran) injection (150-300 IU) based on the follicle-stimulating hormone and anti-Mullerian hormone levels and vaginal ultrasound monitoring. After stimulating the ovaries, 10000 IU of human chorionic gonadotropin (HCG; Iran Hormone Pharmacy, Iran) was injected when 2 follicles with the size of ≥ 18 mm were detected. After 34-36 hr, ultrasound-guided oocyte retrieval was performed under spinal anesthesia.

The fertilized oocytes were investigated after 24 hr. After 3 days, the quality of the embryos was assessed. Participants received 2-3 high-quality embryos. The luteal phase was supported with the daily administration of 400 mg of progesterone suppository rectally and vaginally or 50 mg progesterone ampoules (Iran Hormone Pharmacy, Iran) till the 12 ${}^{\text{th}}$ wk of gestation.

### Outcomes and data collection

The number of clinical pregnancy, miscarriage, preterm delivery, and live birth in both study groups were recorded and compared. Furthermore, the total number of oocytes, meiosis II (MII) oocytes, germinal vesicle oocytes, degenerated oocytes, and the quality of the embryos were evaluated in the 2 groups.

Clinical pregnancy was defined as a fetal cardiac activity in the 6 ${}^{\text{th}}$ -7 ${}^{\text{th}}$ wk of pregnancy. Pregnancy loss earlier than the 20 ${}^{\text{th}}$ wk of gestation was defined as miscarriage. Preterm delivery was defined as the birth of a baby earlier than 37 wk gestation. Live birth was defined as a birth of a live infant ≥ 24 wk of gestation that showed signs of life.

The study was initially designed as a double-blind trial. Participants and physicians who evaluated the outcomes were unaware of the random assignment of individuals to control and intervention groups.

### Ethical considerations

This project was conducted with the permission of the Ethics Committee of the Kurdistan University of Medical Sciences, Sanandaj, Iran (Code: IR.MUK.REC.1397.236). In addition, those who agreed to participate in this study signed awritten. Study participants were assured that their data would remain confidential to researchers.

### Statistical analysis

The data from this research were analyzed through the Statistical Package for the Social Sciences v.21.0 software (SPSS Inc., Chicago, IL, United States), using the Mann-Whitney U test, the student's *t *test, and the Chi-square test. Logistic regression was used to predict the results of ICSI. A p-value < 0.05 was considered as statistically significant.

## 3. Results

**Figure 1:**
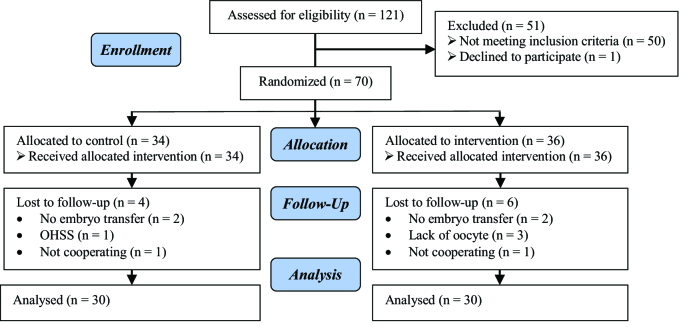
Consort flow diagram for participants involved in the trial. OHSS: Ovarian hyperstimulation syndrome.

Initially, the 121 women were evaluated based on our inclusion criteria. Out of them, 50 women did not meet the inclusion criteria. 1 women was also excluded due to dissatisfaction to continue participating in the study. Finally, 70 women were assigned to 2 groups (36 and 34 women in intervention and control group, respectively). By the end of the study follow-up, 10 women (6 women in intervention and 4 women in control group) were excluded due to dissatisfaction to continue participating in the study or other reasons (not embryo transfer and OHSS). Finally, 60 women (30 women in each group) were remained in the study and entered into analysis (Figure 1).

As shown in table I, there were no significant differences between the 2 groups regarding the demographic characteristics including age, body mass index, familial history of infertility and miscarriage, irregular menstrual cycle, infertility type, and duration of infertility. In addition, the levels of follicle-stimulating hormone, luteinizing hormone, anti-Mullerian hormone, estrogen, and progesterone had no significant differences between the 2 groups (Table I).

Table II indicates that the mean numbers of oocytes, MII oocytes, and 2 pronuclear embryos were significantly higher in the intervention group than in the control group. In addition, the numbers of degenerated oocytes and grade A and B embryos were higher in the intervention group than in the controls. However, these differences were not statistically significant.

Table III shows the results of the pregnancy outcomes in the 2 study groups. The number of clinical pregnancies in the intervention group was significantly higher than in the control group (p = 0.04). The number of women who had live births in the intervention group was also higher than in the control group (p = 0.04). The pregnancy rate in the control group was lower than that of the intervention group. However, this difference was not significant. The miscarriage and preterm delivery rates did not differ between the 2 groups (Table III).

**Table 1:** Comparison of demographic characteristics in 2 study groups (n = 30/each)


	**Control group **	**Intervention group**	**P-value**
Age (yr)*	32.90 ± 4.65	35.67 ± 5.20	0.09
Body mass index (kg/cm^2^)*	24.10 ± 2.24	23.90 ± 2.12	0.07
Familial history of infertility**	7 (23.33)	6 (20.00)	0.30
Familial history of miscarriage**	2 (6.66)	2 (6.66)	0.05
Irregular menstrual cycle**	10 (33.33)	9 (30.00)	0.73
Primary infertility**	20 (66.66)	27 (90.00)	0.73
Secondary infertility**	14 (46.66)	12 (40.00)	0.72
Duration of infertility (yr)*	9.21 ± 4.52	10.57 ± 4.23	0.64
**Follicle stimulating hormone (mIu/ml)^&^**	5.70 ± 1.90	4.48 ± 2.30	0.75
**Luteinizing hormone (mIu/ml)^&^**	5.19 ± 2.03	5.70 ± 1.09	0.74
**Anti-Mullerian hormone (ng/ml)^&^**	5.30 ± 1.11	4.29 ± 1.08	0.65
**Estrogen (ng/ml)^&^**	49.98 ± 5.87	50.72 ± 4.54	0.73
**Progesterone (ng/ml)^&^**	1.71 ± 1.09	1.63 ± 1.27	0.43
* ${}^{,\&}$ Data presented as Mean ± SD, Mann-Whitney U test and Student *t* test, respectively, **Data presented as n (%), Chi-square test and Fisher's exact test			

**Table 2:** Comparison of oocyte quality among the 2 study groups (n = 30/each)


	**Control group **	**Intervention group**	**P-value**
**Oocytes**	6.83 ± 3.36	9.67 ± 3.86	0.04*
**Meiosis II oocytes**	5.43 ± 2.50	7.53 ± 3.71	0.04*
**Germinated vesicle oocytes**	0.37 ± 0.76 (MD = 0, IQR = 0)	0.90 ± 1.39 (MD = 0, IQR = 1)	0.71**
**Degenerated oocytes**	0.80 ± 1.24 (MD = 0, IQR = 1)	0.53 ± 1.04 (MD = 0, IQR = 1)	0.31**
**2 pronuclear embryos**	4.87 ± 2.41	5.17 ± 2.92	0.005*
**Grade A embryos**	3.40 ± 2.37	5.17 ± 2.92	0.10**
**Grade B embryos**	1.37 ± 1.45 (MD = 1, IQR = 2)	1.80 ± 1.34 (MD = 2, IQR = 2)	0.23**
Data presented as Mean ± SD, *Independent sample *t* test, **Mann-Whitney U test, MD: Median, IQR: Interquartile range			

**Table 3:** Comparison of the pregnancy outcomes between the 2 study groups (n = 30/each)


	**Control group **	**Intervention group**	**P-value**
**Not pregnant **	23 (76.66)	13 (43.33)	0.04*
**Clinical pregnancy**	7 (23.33)	17 (56.66)	0.04*
**Miscarriage**	3 (10.00)	6 (20.00)	0.06**
**Preterm delivery**	1 (3.33)	3 (10.00)	0.40**
**Live birth**	3 (10.0)	8 (26.66)	0.04**
Data presented as n (%), *Chi-squared test. **Fisher's exact test			

## 4. Discussion 

In the present study, administration of myo-inositol in women candidates for IVF/ICSI was associated with a higher total number of oocytes and a higher number of M II oocytes. In addition, the clinical pregnancy and live birth rates were also higher in these women.

Infertility imposes a great deal of psychological and financial burden on the cases and their family. A failure of specialized and expensive treatments may result in negative psychological effects on couples. Despite the expansion of experience in ARTs and significant advancements in infertility treatment, the failure of fertility therapy is a major challenge (14). Oocyte quality is the main predictor of the success rate in IVF (15). Various studies have investigated ways for increasing the quality of oocytes and embryos (16, 17). The normal function of the ovaries is of great importance for the health of the reproductive tract. Antioxidant compounds are defensive barriers that maintain the activeoxygen species balance. Disruption of the antioxidant balance can interfere with oocyte maturation, ovulation, fertilization, implantation, and embryo development (17). A study reported that compounds such as Myo-inositol could reduce oxidative stress by enhancing the cellular antioxidant defense (16). The effectiveness of inositol in modifying the process of ovulation has been extensively studied (18, 19).

The findings of a previous study showed that during IVF, pretreatment with Myo-inositol could help to improve the quality of the oocytes and embryos (20). Increased Myo-inositol levels in the follicular fluid can be effective in promoting follicle maturation and the development of high-quality oocytes (18). The results of the current study indicated that Myo-inositol enhanced the total number of oocytes and MII oocytes in women candidates for IVF/ICSI. Similar to our results, a pilot study of a prospective controlled observational trial demonstrated that Myo-inositol could increase the success rate of IVF in poor responders to gonadotropins with low-quality oocytes by increasing the recovery of MII oocytes and ovarian hypersensitivity to gonadotropins (21).

The findings of our study suggested that Myo-inositol affected the maturation of oocytes: it improved the fertilization rate (2 pronuclear embryos) and grade A embryos in the intervention group, and was associated with higher clinical pregnancy and live birth rates as compared with the control group. It should be noted that, except for secondary infertility causes, the 2 groups were homogeneous in terms of causes of infertility and all demographic characteristics (p > 0.05). The heterogeneity related to the control group causes may be due to the differences in using fresh (22 cases in the intervention group and 17 cases in the control group) vs. frozen (n = embryos in the groups (a higher proportion of women in the intervention group used fresh embryos that in the control group). Another reason maybe because of the effect of folic acid on the endometrium and in improving implantation.

The rates of preterm delivery and miscarriage in the intervention group were higher than in the control group. However, in general, most research has shown the beneficial effects of supplements on the outcomes of IVF treatment (21).

A meta-analysis that included 7 articles and 935 subjects, showed promising effects of Myo-inositol in promoting ARTs. Pretreatment with Myo-inositol increased the clinical pregnancy rate by 6.13% and also reduced the miscarriage rate by 27.08%. However, in contrast to these results, we found no significant effect of Myo-inositol in reducing miscarriage in the intervention group. Although the effects of Myo-inositol in infertile women undergoing ICSI or IVF and embryo transfer are promising, the optimal dose of inositol and the type of isomer have not been defined yet. Therefore, the effects of different genera and doses should be considered (22).

The results of this study showed that 4000 mg of Myo-inositol plus 400 mcg of folic acid can improve the rates of clinical pregnancy and live birth in IVF/ICSI cycles. A limited number of articles has shown similar results using 2000 mg. In addition, it has been reported that in women with poor oocyte quality, Myo-inositol and folic acid can improve pregnancy outcomes (23).

## 5. Conclusion

We can conclude that Myo-inositol, an antioxidant compound, can increase the pregnancy success rate in IVF/ICSI cycles, likely by improving the quality of the oocytes and embryos.

##  Conflict of Interest

The authors declare that there are no conflict of interest.
